# Unraveling the Mystery of Hepatic Portal Vein Gas: Exploring Its Benign Nature and Surgical Implications

**DOI:** 10.7759/cureus.41231

**Published:** 2023-06-30

**Authors:** Othman A Iskander

**Affiliations:** 1 Department of Surgery, Jazan University, Jazan, SAU

**Keywords:** gut ischemia, pneumatosis intestinalis, emergency medical service, surgery, portal venous gas

## Abstract

Hepatic portal venous gas (HPVG) is an infrequent yet potentially life-threatening condition that necessitates prompt diagnosis and effective management. This study presents the clinical scenario of an 88-year-old known diabetic patient, with chronic kidney disease (CKD), stroke, and hypertension, who was brought to the emergency department with symptoms of vomiting, constipation, and abdominal pain. Upon conducting a computed tomography (CT) scan of the abdomen, dilatation of the small bowel and pneumatosis intestinalis in the right abdomen, accompanied by the presence of air within the portal vein, were identified. Subsequently, an emergency laparotomy was performed, which revealed no evidence of ischemia, and the patient was treated with IV antibiotics. This case highlights the significance of adopting a multidisciplinary approach and timely interventions in the management of HPVG. The successful resolution of this complex case underscores the importance of prompt diagnosis, appropriate resuscitation, and surgical intervention, all of which play pivotal roles in enhancing patient outcomes.

## Introduction

Hepatic portal venous gas (HPVG) is a condition characterized by the buildup of gas within the portal vein and its branches. This radiological observation is regarded as a serious indicator often linked to life-threatening conditions [1,2]. The discovery of air within the liver has captivated the medical community since its initial identification in infants afflicted by abdominal catastrophe, as documented by Wolf and Evans [3]. It is noteworthy that air can manifest in either the hepatobiliary tree or portal venous system, and its prevalence seems to be more widespread than previously acknowledged.

Presently, owing to the progress made in imaging studies, specifically the extensive utilization of CT scans, the recognition of this condition has become more prevalent [2]. The exceptional sensitivity of CT scans enables them to detect it earlier and more frequently than previous methods [4].

The precise underlying cause of HPVG remains unclear, although various theories have been proposed. One hypothesis suggests that elevated pressure within the intestinal lumen is a key factor in the passage of gas through mesenteric veins to the portal veins [1]. Secondly, the presence of intra-luminal bacteria has been implicated in the production of gas, which subsequently diffuses into the portal venous system [1]. The third theory focuses on mucosal damage resulting from specific disease mechanisms as a potential cause of HPVG. It suggests that in certain pathological conditions, such as gastrointestinal inflammation or necrosis, the compromised integrity of the mucosal lining allows gas to penetrate into the portal venous system [5]. While HPVG was previously primarily associated with intestinal ischemia, it has now been reported in other contexts that may not require surgical intervention for resolution.

## Case presentation

An 88-year-old male presented to the emergency department with complaints of vomiting, abdominal pain, and constipation persisting for the past five days. The patient has a medical history significant for chronic kidney disease, diabetes mellitus, hypertension, and a previous stroke, which has left him paraplegic. He is currently prescribed 75 mg of clopidogrel. Constipation is associated with coffee-ground vomiting. However, he denies fever, weight loss, changes in urine or stool color, or loss of appetite.

Upon physical examination, the patient exhibited consciousness, alertness, and orientation. Vital signs were within normal range, temperature of 36.6°C, blood pressure measuring 118/76 mmHg, respiratory rate of 18 breaths per minute, and a pulse rate of 88 beats per minute. The abdomen appeared symmetrically distended, but no tenderness, rigidity, or abdominal masses were observed. Bowel sounds were normal and digital rectal examination yielded normal results. Laboratory investigations are shown in Table 1.

**Table 1:** Summary of laboratory investigations and reference values.

Laboratory investigations	Observed value	Reference value
Hemoglobin	15.2 g/dL	13.5-17.5 g/dL
Leukocyte count	13.5 × 10^3^/μL	4.5-11.0 × 10^3^/μL
Alkaline phosphatase	593 U/L	40-126 U/L
Arterial blood gas (ABG)	7.32	pH: 7.35-7.45
Prothrombin time	30 s	11-13 s
Activated partial thromboplastin time (APTT)	52 s	25-35 s
International normalized ratio (INR)	2.21	0.8-1.2
Urea level	191 mmol/L	2.5-7.1 mmol/L
Creatinine level	218 mmol/L	53-115 μmol/L
Plasma sodium level	134 mmol/L	135-145 mmol/L
Plasma potassium level	3.91 mmol/L	3.5-5.0 mmol/L
Serum albumin level	41 g/dL	35-50 g/L
Total bilirubin level	24 μmol/L	3.4-17.1 μmol/L
Total protein level	79 g/dL	60-80 g/L
Gamma-glutamyl transferase (GGT) level	57.2 U/L	0-51 U/L

**Figure 1:**
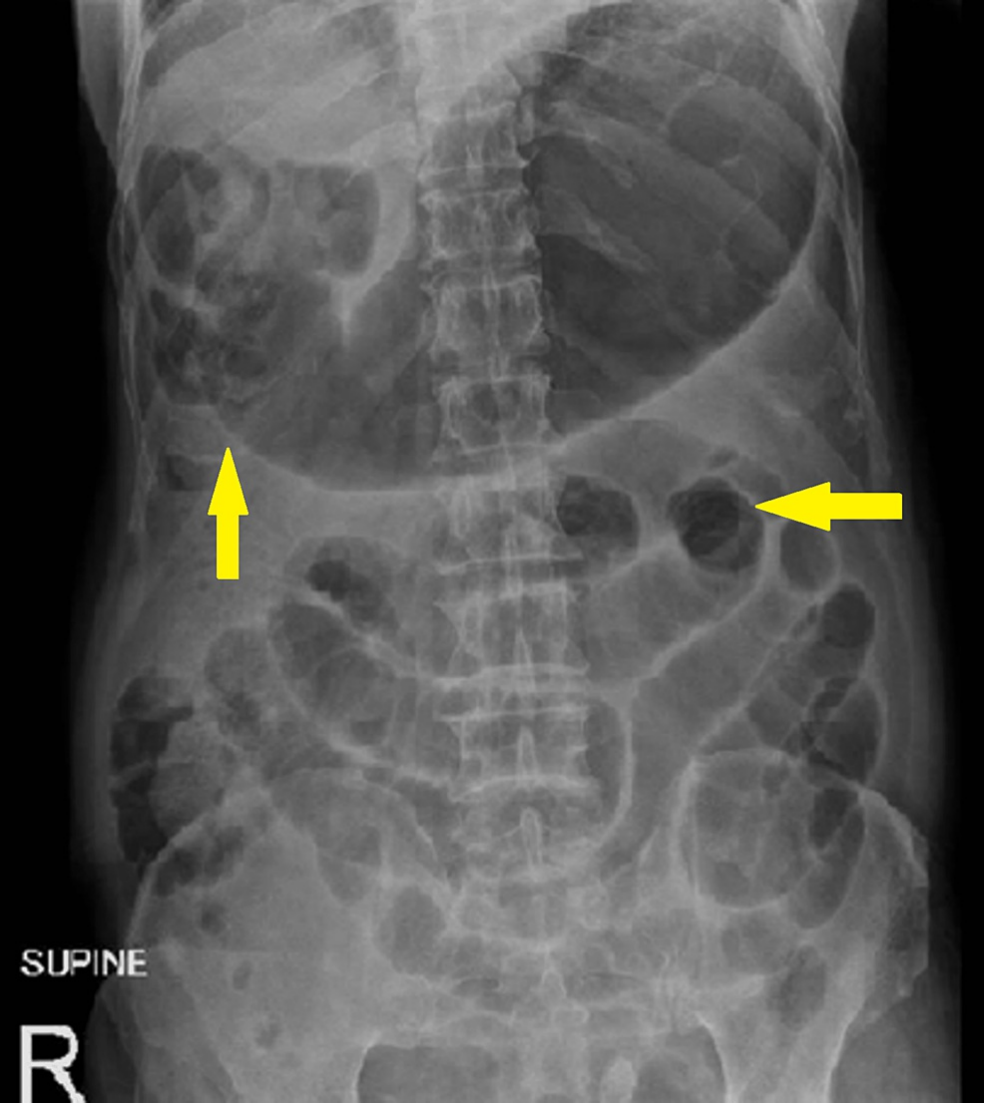
Abdominal supine x-ray demonstrating significant gastric distension and multiple dilated small bowel loops.

**Figure 2:**
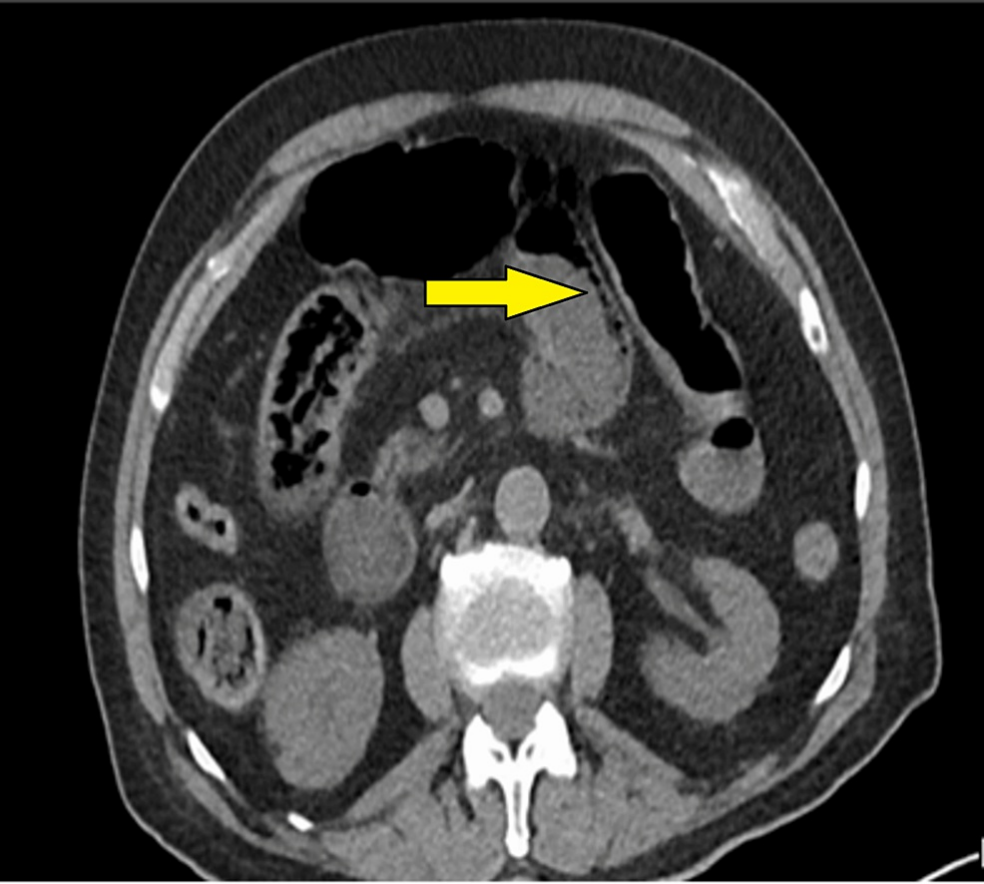
CT scan of the abdomen showing the presence of air within the small bowel segment (pneumatosis intestinalis).

**Figure 3:**
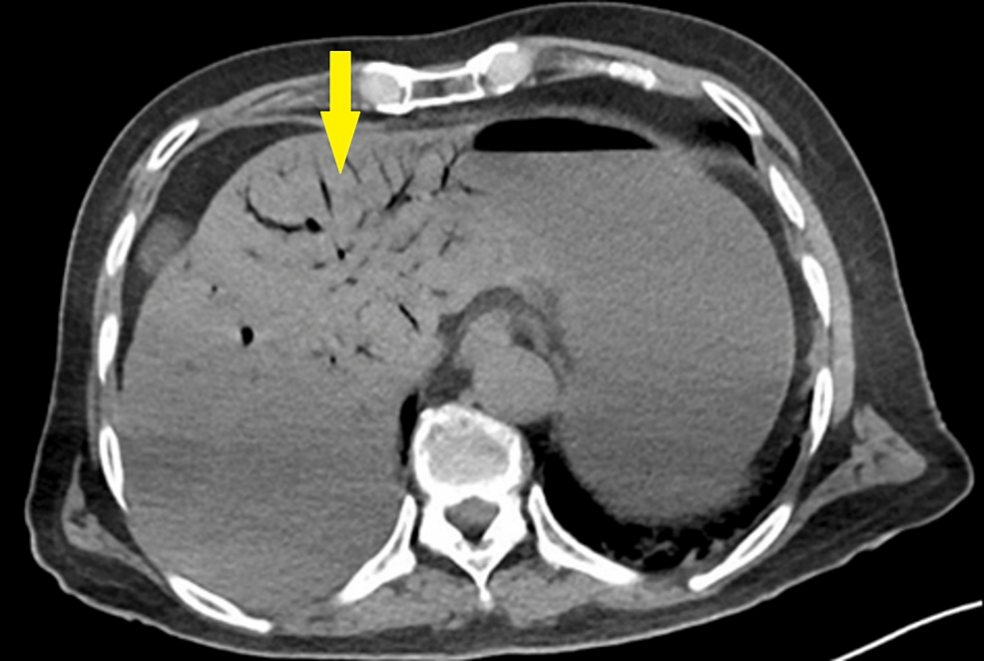
CT scan of the abdomen revealing the presence of air within the portal vein, predominantly observed on the right side of the liver.

Radiographic findings showed multiple dilated small bowel loops and marked gastric distension without air beneath the diaphragm on a supine abdominal x-ray (Figure 1). A non-contrast CT scan of the abdomen (due to chronic kidney disease) revealed dilatation of the small bowel and pneumatosis intestinalis in the right abdomen (Figure 2), along with the presence of air in the portal vein (Figure 3). Consequently, the patient was promptly transferred to the operating room for an emergency exploratory laparotomy under general anesthesia.

**Figure 4:**
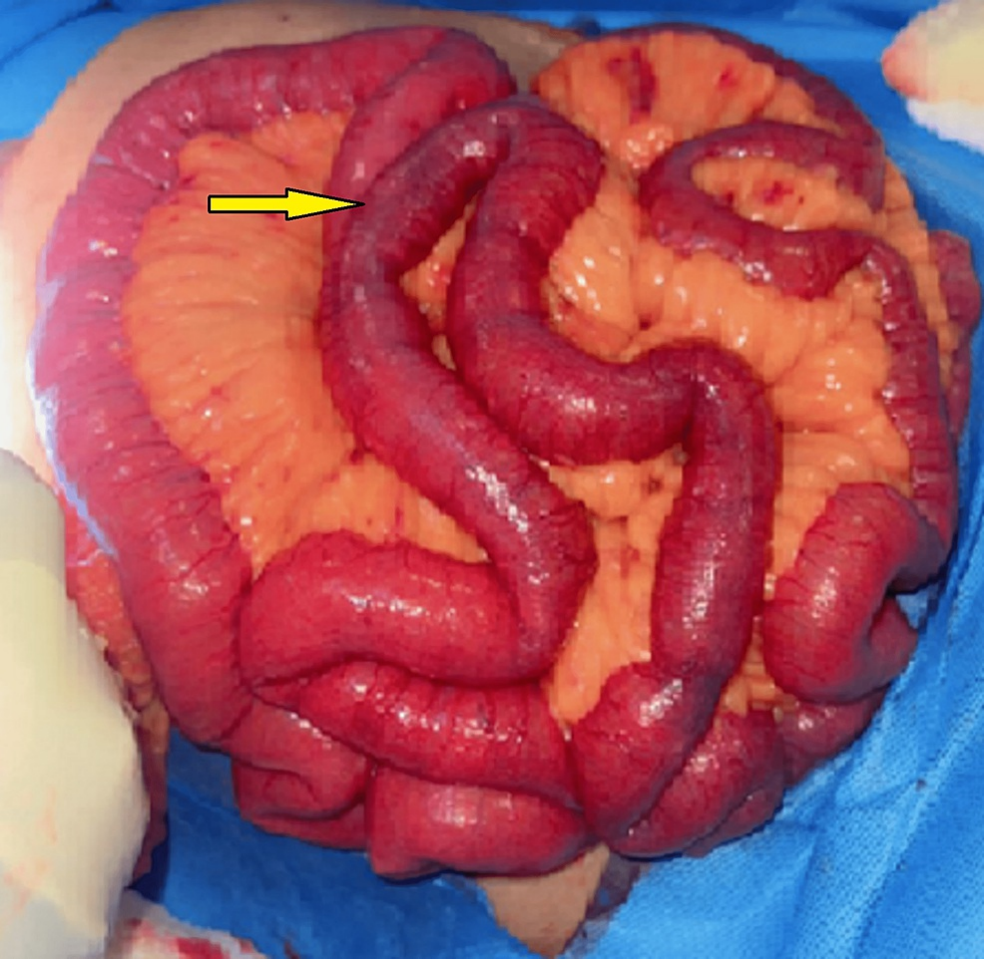
Intra-operative image demonstrating visually normal-looking bowels during laparotomy.

During the diagnostic laparotomy, a comprehensive examination of the abdominal cavity was conducted, revealing no signs of small bowel or large bowel ischemia. Furthermore, the stomach, liver, and spleen demonstrated adequate vascularization (Figure 4).

The patient's abdomen was subsequently closed, and he was transferred to the ward for post-operative care. Empirical antibiotics and fluid therapy were administered, resulting in gradual improvement. The patient was discharged on the fifth-day post-surgery.

## Discussion

In 1955, Wolfe and Evans initially documented the occurrence of gas in the intra-hepatic portal system among children diagnosed with necrotizing enterocolitis [6]. This discovery was later reported in adult patients by Susman and Senturia five years later [7]. Previously, the primary cause of PVG was bowel ischemia [8]. Given the strong association between PVG and severe intestinal ischemia, its presence is now regarded as a foreboding indication.

Through continuous analysis of HPVG (hepatic portal venous gas) cases over the span of three decades, it has become increasingly evident that HPVG is an imaging manifestation resulting from various diseases. Based on a retrospective analysis of previous literature, Bloom et al. concluded that a significant proportion of cases were associated with intestinal ischemia resulting in a mortality rate of more than 90% [9]. This highlights the worse prognosis commonly observed in HPVG cases related to ischemic intestinal necrosis [9]. More recently, Koizumi et al. performed a statistical examination of the Japanese inpatient database and found that 53% of patients diagnosed with HPVG had underlying conditions related to intestinal ischemia [2]. Additionally, they reported an in-hospital mortality rate of 27.3%. Among the patients, 32% underwent surgical intervention, resulting in a notable decrease in mortality rate. These findings align closely with the research conducted by García-Moreno et al. [10].

The occurrence of HPVG is thought to be related to the following three possible mechanisms: mucosal damage, bowel distension, and sepsis resulting from gas-forming bacteria which was in our case. In more than two-thirds of patients diagnosed with hepatic portal venous gas, a necrotic bowel was observed. Furthermore, the presence of pneumatosis intestinalis (PI), which is characterized by the presence of gas-filled cysts in the subserosal and submucosal layers of the gastrointestinal tract, frequently coincided with hepatic portal venous gas [11,12].

The diagnosis of HVPG (hepatic portal venous gas) is typically accomplished through radiological examinations, such as x-ray abdomen or computed tomography (CT). Among these imaging techniques, CT is considered the preferred method due to its superior sensitivity in detecting HVPG, making it the gold standard for diagnosis [13]. In around 80% of cases, the presence of portal air cannot be visualized through abdominal plain films. However, there are instances where plain abdominal films may reveal signs of pneumatosis intestinalis, specifically affecting either the large or small bowel. While abdominal plain films may not consistently detect portal air, they can occasionally offer indications of pneumatosis intestinalis in specific bowel segments [14]. CT scans and ultrasound have higher sensitivity in visualizing smaller quantities of portal venous gas when compared to plain radiographs.

The treatment approach for HPVG is primarily determined by the underlying cause, as well as the presence or absence of peritonitis or intestinal perforation, and the overall condition of the patient. In the case of our patient, whose condition was deteriorating, immediate measures were taken to stabilize him. Intra-venous fluid resuscitation and administration of antibiotics were initiated, and an emergency explorative laparotomy was performed, but no sign of ischemia was noticed.

## Conclusions

In conclusion, this case serves as a compelling reminder of the significance of adopting a multidisciplinary approach and timely interventions in the management of HPVG. Prompt diagnosis, appropriate resuscitation, and surgical intervention are vital components that contribute to the successful resolution of this complex condition. By recognizing the importance of collaboration and acting swiftly, healthcare professionals can significantly enhance patient outcomes and improve overall prognosis in cases of HPVG.

## References

[1] Abboud B, El Hachem J, Yazbeck T, Doumit C (2009). Hepatic portal venous gas: physiopathology, etiology, prognosis and treatment. World J Gastroenterol.

[2] Koizumi C, Michihata N, Matsui H, Fushimi K, Yasunaga H (2018). In-hospital mortality for hepatic portal venous gas: analysis of 1590 patients using a Japanese National Inpatient Database. World J Surg.

[3] Kesarwani V, Ghelani DR, Reece G (2009). Hepatic portal venous gas: a case report and review of literature. Indian J Crit Care Med.

[4] Griffiths DM, Gough MH (1986). Gas in the hepatic portal veins. Br J Surg.

[5] Liebman PR, Patten MT, Manny J, Benfield JR, Hechtman HB (1978). Hepatic-portal venous gas in adults: etiology, pathophysiology and clinical significance. Ann Surg.

[6] Wolfe JN, Evans WA (1955). Gas in the portal veins of the liver in infants; a roentgenographic demonstration with postmortem anatomical correlation. Am J Roentgenol Radium Ther Nucl Med.

[7] Susman N, Senturia HR (1960). Gas embolization of the portal venous system. Am J Roentgenol Radium Ther Nucl Med.

[8] Najafian H, Habibi M, Reilly T (2003). Hepatic portal vein gas: clinical features and outcomes. Am Surg.

[9] Bloom RA, Lebensart PD, Levy P, Craciun E, Anner H, Manny J (1990). Survival after ultrasonographic demonstration of portal venous gas due to mesenteric artery occlusion. Postgrad Med J.

[10] García-Moreno F, Carda-Abella P (2007). Hepatic portal venous gas. Ann Hepatol.

[11] Wang M, Song J, Gong S, Yu Y, Hu W, Wang Y (2020). Hepatic portal venous gas with pneumatosis intestinalis secondary to mesenteric ischemia in elderly patients: two case reports. Medicine (Baltimore).

[12] Ito M, Horiguchi A, Miyakawa S (2008). Pneumatosis intestinalis and hepatic portal venous gas. J Hepatobiliary Pancreat Surg.

[13] Kinoshita H, Shinozaki M, Tanimura H (2001). Clinical features and management of hepatic portal venous gas: four case reports and cumulative review of the literature. Arch Surg.

[14] Yip CK, Ng VK, Man DW, Metreweli C (1990). Sonographic recognition of pneumatosis intestinalis and portal gas in an 11-months-old infant - case report. Australas Radiol.

